# Chemical-induced disease relation extraction via convolutional neural network

**DOI:** 10.1093/database/bax024

**Published:** 2017-04-02

**Authors:** Jinghang Gu, Fuqing Sun, Longhua Qian, Guodong Zhou

**Affiliations:** 1 School of Computer Science and Technology, Soochow University, 1 Shizi Street, Suzhou, China; 2 Department of Gynecology Minimally Invasive Center, Beijing Obstetrics and Gynecology Hospital, Capital Medical University, 17 Qihelou Street, Beijing, China

## Abstract

This article describes our work on the BioCreative-V chemical–disease relation (CDR) extraction task, which employed a maximum entropy (ME) model and a convolutional neural network model for relation extraction at inter- and intra-sentence level, respectively. In our work, relation extraction between entity concepts in documents was simplified to relation extraction between entity mentions. We first constructed pairs of chemical and disease mentions as relation instances for training and testing stages, then we trained and applied the ME model and the convolutional neural network model for inter- and intra-sentence level, respectively. Finally, we merged the classification results from mention level to document level to acquire the final relations between chemical and disease concepts. The evaluation on the BioCreative-V CDR corpus shows the effectiveness of our proposed approach.

**Database URL:** http://www.biocreative.org/resources/corpora/biocreative-v-cdr-corpus/

## Introduction

Automatically understanding chemical–disease relations (CDRs) is crucial in various areas of biomedical research and health care (1–3). Although some well-known manual curation efforts like the Comparative Toxicogenomics Database (CTD) project (4, 5) have already curated thousands of documents for CDRs, the manual curation from literature into structured knowledge databases is time-consuming and insufficient to keep up to date.

Due to the high cost of the manual curation, several attempts have been made on automatic biomedical information extraction with some promising results using text-mining technologies (6–9). However, many tasks such as identifying biomedical concepts (10, 11) and extracting relations between biomedical entities (12), still remain challenging.

To this end, the BioCreative V (BC5) community proposed a challenging task of automatic extraction of CDRs from biomedical literature, which was aimed to encourage research on text mining in this area. The task consisted of two subtasks: the disease named entity recognition task and the chemical-induced disease (CID) relation extraction task. The first was to identify diseases and normalize them to corresponding Medical Subject Headings (MeSH) (13) concept identifiers and the second was to identify causal relations between chemicals and diseases denoted by MeSH identifier pairs. In this paper, we mainly focus on the CID relation extraction task.

Different from previous biomedical relation extraction tasks such as disease-gene association (6, 7) and protein–protein interaction (8, 9), the CID relations are determined at document level, i.e. the relations could be described across sentences (14). Furthermore, the CID subtask required the relations hold between the most specific diseases and chemicals.

Since chemical and disease entities may have multiple mentions spanning sentences in a document, we regard the case as ‘intra-sentence level’ when mentions of chemical and disease occur in the same sentence, or as ‘inter-sentence level’ otherwise. Thus, the CID relation extraction task can be simplified from document level to mention level, taking the following sentences into consideration:

- Possible intramuscular midazolam-associated cardiorespiratory arrest and death.
- Midazolam hydrochloride is commonly used for dental or endoscopic procedures.
- Although generally consisted safe when given intramuscularly, intravenous administration is known to cause respiratory and cardiovascular depression.
- This report describes the first published case of cardiorespiratory arrest and death associated with intramuscular administration of midazolam.
- Information regarding midazolam use is reviewed to provide recommendation for safe administration.

Above sentences are extracted from the same document (PMID: 2375138). Among them, the texts in ***bold*** are mentions of chemicals and diseases, where ***midazolam*** and ***Midazolam hydrochloride*** refer to the same chemical concept whose identifier is D008874 (C1), ***cardiorespiratory arrest*** represents a disease concept whose identifier is D006323 (D1), ***respiratory and cardiovascular depression*** refers to a disease concept whose identifier is D012140 (D2), and ***death*** refers to another disease concept with the identifier of D003643 (D3). The chemical C1 has two intra-sentence level co-occurrences with the disease D1 in both sentences (*a*) and (*d*), while it has an inter-sentence level co-occurrence with the disease D2. However, not all occurrences of chemicals and diseases are considered as a valid CID relation. For instance, according to the task guidelines (15), there should be no relation between C1 and D3 because the concept of ***death*** is too general to reflect a CID relation.

Since relation extraction task is usually considered as a classification problem, various statistical machine-learning approaches have been successfully applied to the CID task. Jiang *et al.* (16) used a logistic regression model with linguistic features to extract CID relations. Zhou *et al.* (17) applied a kernel-based support vector machine (SVM) method for the CID task by capturing syntactic associations between chemicals and diseases. Our previous work (18, 19) proposed a model incorporating different maximum entropy (ME) classifiers with rich linguistic information including various lexical and syntactic features to extract CID relations at intra- and inter-sentence level, respectively. In addition, methods using prior knowledge have been proved to be effective for the CID relation extraction task. Xu *et al.* (20) fed abundant knowledge-based features into two different SVM classifiers at sentence level and document level, respectively, and they obtained the top performance during the BC5 online evaluation. Pons *et al.* (21) employed rich features derived from various knowledge databases for an SVM classifier to extract CID relations. Particularly, Peng *et al.* (22) proposed a hybrid system for the CID task achieving the state-of-the-art performance. They adopted an SVM model with a rich set of features including statistical, linguistic and various domain knowledge features. Furthermore, they augmented more external training data in order to further improve the performance.

Recently, on the new benchmark dataset of SemEval-2010 Task 8 (23) on relation classification task, deep neural networks (24) such as convolutional neural network (CNN) have exhibited remarkable potential (25–27) on account of such methods providing an automatic way of feature representation without much manual efforts on feature engineering. Zeng *et al.* (25) presented a CNN paradigm combining lexical features with position features to perform relation classification. They obtained an *F*-score of 82.7% on the SemEval-2010 Task 8 dataset while the best performance of the traditional classifier, i.e. SVM, only achieved 82.2%. Nguyen and Grishman (27) employed a CNN-based model utilizing multiple sizes of filters to conduct the relation classification task, and they achieved an *F*-score of 82.8%. Santos *et al.* (26) proposed a ranking-based CNN architecture to perform the relation classification task. They employed a novel pairwise ranking loss function and achieved the state-of-the-art performance with an *F*-score of 84.1% on the benchmark dataset.

With respect to deep neural networks, recurrent neural network (RNN) serves as another widely exploited model that has been shown to be competitive in relation classification tasks. Zhang and Wang (28) employed a bi-directional RNN framework to learn long-distance relation patterns to tackle the relation classification problem, and they obtained an *F*-score of 82.5% on the SemEval-2010 Task-8 dataset. Xu *et al.* (29) proposed to use a variant of RNN, i.e. long short-term memory (LSTM) network, to identify relations. They employed the LSTM network to pick up the semantic information in the shortest dependency paths and finally achieved an *F*-score of 83.7%. In the same vein, Zhou *et al.* (30) proposed a neural network framework for the CID relation extraction task for the first time. They designed a hybrid system combining an LSTM network with a kernel-based SVM model. In their method, the SVM model was designed to capture the syntactic features and the LSTM was intended to grasp the potential semantic representations, respectively.

Different from RNN, which is prone to learn from long word sequences, CNN is demonstrated to consistently extract local features due to its elegant characteristic of capturing the most useful features in a flat structure as well as representing them in an abstract way effectively. In most cases, relations are predominantly reflected in local feature rather than global word sequence, and the popularity of the shortest dependency path of relation extraction demonstrates that local information in dependency context is more useful for identifying relations. However, there are few works on taking advantage of CNNs for biomedical information extraction, especially for the CID relation extraction task. We therefore proposed a CNN-based model to learn a more robust relation representation based on both sentences and dependency paths for the CID relation extraction task, which could naturally characterize the relations between chemical and disease entities.

In this paper, we present our approach for the CID relation extraction subtask of the BioCreative-V CDR task. We improve our previous work (18) by adopting a CNN-based model at intra-sentence level. Our primary goal was to develop a machine learning (ML) method with good robustness and generalization ability which could be applied to various relation extraction tasks. We first extracted CID relations at mention level by using a ME model with linguistic features for inter-sentence level, and a convolutional neural network model with multi-level semantic features for intra-sentence level, respectively. Then we merged the results of both levels to obtain CID relations between entity concepts at document level. In addition, the hypernym relationship between entities was taken into consideration during the training stage for constructing more precise training instances as well as during the testing stage for filtering the extracted instances in order to improve the extraction performance. Heuristic rules were finally adopted in the post-processing (PP) stage to further improve the performance.

To the best of our knowledge, this is the first time to model the CID relation extraction problems with a convolutional neural network on dependency information. The experimental results on the CDR corpus show the effectiveness of our proposed approach.

## Materials and methods

In this section, we first present a brief introduction to the CDR corpus, then we systematically describe our approach for the CID relation extraction task.

### Dataset

The CDR corpus contained a total number of 1500 MEDLINE articles (only titles and abstracts) (14) that were further divided into three subsets: the training, development and test sets. All the articles were manually annotated with chemicals, diseases and CDRs using the MeSH concept identifiers. In particular, the CDR relations were annotated per pair of chemical and disease concept identifiers in a document rather than per pair of entity mentions. Table 1 reports the statistics on the numbers of articles and relations in the corpus.

**Table 1.** The CID relation statistics on the corpus

Task datasets	No. of articles	No. of CID relations
Training	500	1038
Development	500	1012
Test	500	1066

It is worth noting that the CDR corpus was generated from the CTD knowledge database the construction of which was an enormous curation project lasting for decades. In addition, the inter-annotator agreement (IAA) of the CID relations is unknown. Wiegers *et al.* (31) reported a surrogate IAA score of 77% for annotation of chemical–gene interactions in the CTD corpus and this IAA score may presumably approximate the agreement of the CID relation annotation. However, formal assessment of IAA on the CID relations still needs to be performed.

### Method

**Figure 1.**
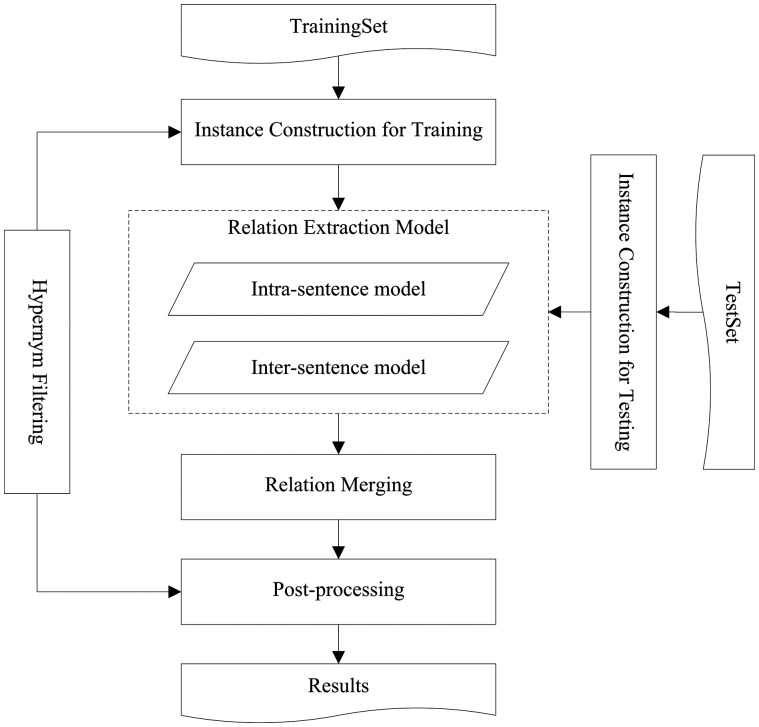
The system workflow diagram.

Figure 1 depicts the architecture of our ML system. It first extracts CID relations using a ME classifier for inter-sentence level and a convolutional neural network for intra-sentence level, respectively. Then it merges the classification results from both levels to obtain relations at document level. Finally, simple yet effective heuristic rules are applied in the PP stage to find the most likely relations in the documents where none relation can be identified by our system. Additionally, since the CID task requires the most specific relations between entities, a hypernym filtering module is adopted during both training and testing stages to obtain more accurate classification models and better extraction performance, respectively.

Different from (18), we improve the previous work by adopting a novel convolutional neural network at intra-sentence level and heuristic rules in the PP stage to promote the performance. The whole process can be divided into six sequential steps as follows.

#### Relation instance construction

Prior to relation extraction, relation instances for both training and testing stages should be first constructed. All the instances are generated from chemical and disease mentions in a pairwise way following (18). The instances are then pooled into two groups at intra- and inter-sentence level, respectively. The former means a mention pair is within the same sentence, while the latter means otherwise.

#### Hypernymy filtering

The CID task is aimed to automatically extract the relations between the most specific diseases and chemicals, that is, the relations between hyponym concepts should be considered rather than between hypernym concepts. However, in some cases, there is a hypernymy/hyponymy relationship between concepts of diseases or chemicals, where a concept is subordinate to another more general concept. Thus, it is possible that despite some pairs of entities expressing the positive relations, their relation instances should still be taken as negative because they could not exactly express the most specific CID relations, leading to degrading the performance. Therefore, we leverage the MeSH tree numbers of concepts to determine the hypernymy relationship between entities in a document and remove those negative instances that involve entities which are more general than other entities already participating in the positive ones. More details can be found in the previous work (18).

#### Relation extraction at inter-sentence level

The CID relation extraction at inter-sentence level can be recast as a binary classification problem. The training instances are fed into a learner to derive a classification model which is in turn used to predict the relation for the test instances. More details of this step can be found in the previous work (18).

#### Relation extraction at intra-sentence level

**Figure 2.**
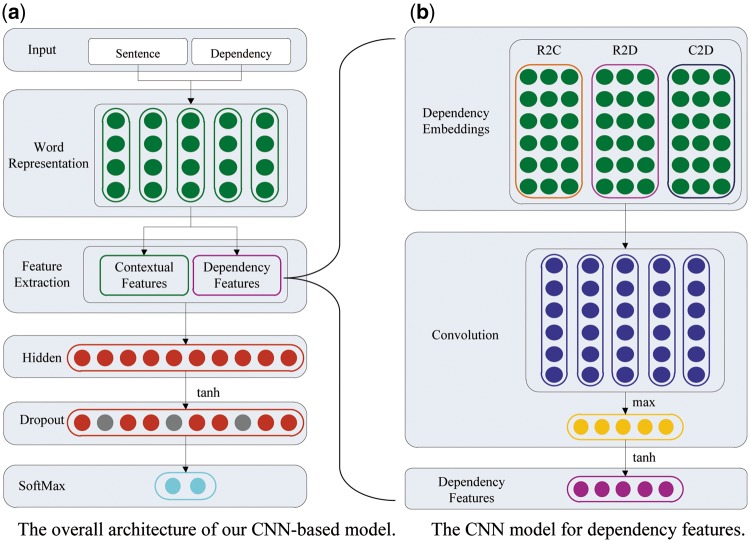
The architecture of our CNN-based model for the intra-sentence level relation extraction. (a) The overall architecture of our CNN-based model; (b) the CNN model for dependency features.

Figure 2 presents the architecture of our CNN-based neural network for the intra-sentence level relation extraction. As depicted in Figure 2a, the model takes as input sentences with marked entity mentions, together with dependency paths. The model can discover multiple levels of features, where higher levels represent more abstract aspects of the input. It primarily consists of five layers as follows: *Word Representation*, *Feature Extraction*, *Hidden*, *Dropout* and *SoftMax*.

Our CNN-based model first converts each token in the input sequences (sentences or paths) into a word embedding vector, then extracts contextual features from sentences and dependency features from dependency paths, respectively. Afterwards, the model concatenates both contextual features and dependency features into a vector, and sends this vector to the following hidden layer to obtain the final feature vector characterizing more abstract representation. Finally, the feature vector is fed into a softmax layer to compute the ultimate confidence scores for relation labels. During the training process, a dropout operation is employed upon the feature vector after the hidden layer in order to address the over-fitting problem. The final output of the CNN-based model is a vector, whose dimension is equal to the number of the predefined relation types and the value of each dimension is the confidence score of the corresponding relation type.

##### (1) Word representation

With the intention of generating more meaningful representation of tokens, neural networks require converting tokens into low-dimensional dense vectors. For this purpose, a *Word Representation* layer is used to transform each token in the input sequence into a fixed-length word embedding through a look-up table.

Generally, each token in the look-up table is associated with an index and each sentence is an array of indices. Given an input sequence *s* consisting of *n* token indices *s *= [*t*_1_, *t*_2_,…, *t_n_*], a token *t_i_*can be represented by a *d*_0_-dimensional real-valued word embedding vector ***e***_*i*_, which is obtained by looking up in a corresponding vocabulary table $T\in R^{d_{0}\times |V|}$, where *V* is a fixed-sized vocabulary and |*V*| is the size of *V*. Each column vector in ***T*** corresponds to a word embedding. Therefore, the original input sequence *s* can be viewed as a matrix **x** of size *d*_0_ × *n* that concatenates the word embedding of each token in the order of their occurrence, that is, *s* is represented by **x** = [***e*_1_**, ***e*_2_**,…, ***e_n_***]. We transform a token *t_i_*into its word embedding ***e****_i_*by using the matrix–vector product:

(1) $$e_{i}=Tu_{t_{i}}$$

where $u_{t_{i}}$ is a vector of size |*V*| which has value 1 at index *t_i_* and zero at all other positions. The vocabulary table ***T*** is a parameter to be learned, while the size of the word embedding *d*_0_ is a hyper-parameter.

Since CNN can only work with fixed-length input, we should first augment all the input sentences to be of the same length by appending special padding words to the short ones before sending them into the *Word Representation* layer. The padding word we used is denoted by ‘*<PAD>*’, whose index in ***T*** represents the vector filled with zeros.

##### (2) Feature extraction

In *Feature Extraction*, both contextual features and dependency features are taken into account to learn the more abstract representation of relations. The details are shown as follows:

###### (i) Contextual features

Despite the meaningful representation of word embedding, word vectors are still less informative for relation extraction. Thus, we take the contextual features around the target mentions into consideration as they can offer important cues for determining relations.

In this paper, the contextual feature is generated by concatenating multiple vectors, which consists of the word embeddings of the mentions, the word embeddings of the tokens around the mentions within an odd window size of *w*, and the word embeddings of the verbs between the mentions. Since relations in the biomedical literature are usually expressed in a predicative form, we believe verbs also play an important role in relation extraction. All of the above embeddings are concatenated to serve as the contextual feature vector ***c***. Table 2 presents the selected word embeddings that are related to the contextual features.

**Table 2.** The contextual features

No.	Features
L1	Chemical mention
L2	Disease mention
L3	(*w*−1)/2 left and right tokens of chemical mention
L4	(*w*−1)/2 left and right tokens of disease mention
L5	Verbs in between

Note that some entity mentions may have more than one token, we thus take the mean value of all word embeddings of the tokens within a mention to represent the corresponding mention embedding. In addition, the number of verbs between mentions varies in different sentences, we therefore pad the sequences of verbs to be of the same length by repetitively appending the padding word to the short ones.

###### (ii) Dependency features

Apart from the contextual features, we argue that the dependency associations can offer more concise and effective information to determine CID relations between entity mentions as in other domains (29). Figure 2b illustrates the architecture of our CNN model to extract the dependency features. We first concatenate the embeddings of dependency paths into an embedding matrix, then we convolute the matrix with a max-pooling operation to encode the dependency features in a more abstract way. In our model, three dependency paths are taken into account: the dependency path from root to chemical (R2C), the dependency path from root to disease (R2D) and the dependency path from chemical to disease (C2D). For instance, take the following sentence into consideration:

f. The dipyridamole induced his hyperemia.

**Figure 3.**
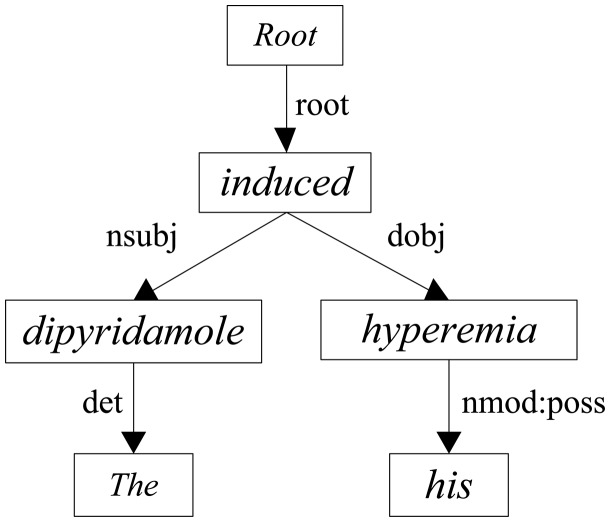
The dependency parsing tree of the example sentence.

Figure 3 exhibits the dependency parsing tree of sentence *(f)* where *dipyridamole* is the mention of the chemical and *hyperemia* is the mention of the disease. Table 3 shows the corresponding directed dependency paths of R2C, R2D and C2D, respectively.

**Table 3.** The dependency paths of the example sentence

Name	Dependency paths
R2C	ROOT↓root↓induced↓nsubj↓dipyridamole
R2D	ROOT↓root↓induced↓dobj↓hyperemia
C2D	dipyridamole↑nsubj↑induced↓dobj↓hyperemia

Obviously, as the input of the CNN model, each dependency path can be regarded as a special ‘sentence’ which should be padded into the same length as well. The ‘tokens’ in a path consists of words, dependency relation tags, and dependency directions.

Similar to other CNN-based models (25, 26), we also consider a fixed size window of tokens around the current token to capture its local features in a dependency path. The window size is set to an odd number *v*, indicating that there are (*v *– 1)/2 tokens before and after the candidate token, respectively. Assuming each path of *l* length, the combination of the dependency paths is then transformed into the matrix $X_{0}\in R^{vd_{o}\times 3l}$ according to the look-up table ***T***. For example, when we set *v*=3, the representation of the third word *induced* in the C2D is expressed as [‘↑’, ‘induced’, ‘↓’]. Similarly, considering the whole sentence, the whole C2D path can be represented as follows: {[‘<PAD>’, ‘dipyridamole’, ‘↑’], [‘dipyridamole’, ‘↑’, ‘nsubj’],…, [‘↑’, ‘hyperemia’, ‘<PAD>’]}.

###### (iii) Convolution

To predict a relation, it is necessary to acquire more abstract features of all local features. With respect to neural networks, the convolutional approach is a natural way to merge all of the features and select the most informative ones. Similar to Collobert *et al.* (24), after being fed into the convolutional layer, the matrix ***X***_0_ is processed with the convolution operation:

(2) $$Z=W_{1}X_{0}+b_{1}$$

where $W_{1}\in R^{n_{1}\times vd_{0}}$ is the parameter matrix, $b_{1}\in R^{n_{1}}$ is the bias term and *n*_1_ is the hyper-parameter. We can see that the convoluted features are able to greatly reduce the number of the parameters to be learned. After the linear transformation is applied, the output $Z\in R^{n_{1}\times 3l}$ would represent the features more abstractly.

A pooling operation is then utilized to further abstract the features generated from the convolution operation preserving the most useful information. The popular pooling function is *max* because of its responsibility for identifying the most important or relevant features. The max pooling operation on ***Z*** can be written as:

(3) $$m_{i}=\text{max}\;Z(i,\;\cdot )\;0\leq i\leq n_{1}$$

where ***Z***(*i*, ⋅) denotes the *i*th row of matrix ***Z***. After the max-pooling operation, we obtain the dependency feature vector $p=\{m_{1},m_{2},\ldots ,m_{n_{1}}\}$, the dimension of which is no longer related to the path length.

Afterwards, we send the dependency feature vector ***p*** through a non-linear transformation to obtain the final dependency feature vector. We take the non-linear transformation of hyperbolic tan* h* as the activation function. Formally, the non-linear transformation can be written as:

(4) d=tan  h(p)

where $d\in R^{n_{1}}$ can be considered as the final dependency feature vector with a higher level representation.

##### (3) Hidden

The automatically learned contextual and dependency features mentioned above are concatenated into a single vector ***k* = [*c***, ***d*]**, where $k\in R^{n_{f}}$ (*n_f_* equals *n*_1_ plus the dimension of the contextual features), which is then fed into a hidden layer. The hidden layer applies linear and non-linear transformations on ***k*** to obtain the final feature vector ***r***. The transformations can be written as:

(5) $$r=\text{tan}\;\;h(W_{2}k+b_{2})$$

where $W_{2}\in R^{n_{2}\times n_{f}}$ is the transformation matrix, $b_{2}\in R^{n_{2}}$ is the bias term and *n*_2_ is the hyper-parameter. The vector $r\in R^{n_{2}}$ is the output of the hidden layer.

##### (4) Dropout

During the training step, before actually applying the feature vector ***r***, we conduct a dropout operation on ***r*** to prevent the over-fitting problem of the hidden units by randomly setting the elements of ***r*** to zeros by a proportion *p* and generate the vector ***h*** accordingly:

(6) h=r ∘ m

where·is an element-wise multiplication and ***m*** is a mask vector whose elements follow the Bernoulli distribution with the probability *p*. However, the feature vector***r*** is not dropped out during the testing step. The dropout vector ***h*** is then fed into the softmax layer at the end to perform relation classification.

##### (5) Softmax

To compute the confidence of each relation, the vector $h\in R^{n_{2}}$ is fed into the softmax layer.

(7) $$o=W_{3}h+b_{3}$$

where $W_{3}\in R^{n_{3}\times n_{2}}$ is the transformation matrix $b_{3}\in R^{n_{3}}$ is the bias term and $o\in R^{n_{3}}$ is the final output of the network. The value *n*_3_ equals to the number of the predefined relation types for the classification. Each output can be then interpreted as the confidence score of the corresponding relation. This score can be interpreted as a conditional probability by applying a softmax operation.

To learn the parameters of the network, we use the predicted labels of ***o*** and the gold annotation labels in the training set by adopting the following objective function:

$$J(\theta )\in -\frac{1}{m}\sum_{i=1}^{m}\;\text{log}\;p(y_{i}|x_{i},\theta )+\lambda ||\theta |{|}^{2}$$

where *p*(*y_i_*|*x_i_*,*θ*) is the confidence score of the golden label *y_i_* of the training instance *x_i_*, *m* is the number of the training instances, *λ* is the regularization coefficient and *θ* = {***T***, ***W***_1_, ***b***_1_, ***W***_2_, ***b***_2_, ***W***_3_, ***b***_3_} is the set of parameters.

#### Relation merging

After relation extraction, we merge the results of the two separate mention levels to obtain the final relations between chemicals and diseases at document level. Since a pair of entities may have multiple mention pairs at intra- or inter-sentence level, we assume that if there is at least one pair of the mentions could explicitly support the CID relation, we believe there is a true CID relation between the entities. More details can be found in the previous work (18).

#### Post-processing

When no CID relations can be identified in an abstract, the following heuristic rules are applied to find the most likely relations:

- All chemicals in the title are associated with all diseases in the entire abstract.
- When there is no chemical in the title, the most-frequently mentioned chemical in the abstract is associated with all diseases in the entire abstract.

## Experiments and results

In this section, we first present our experiment settings, then we systematically evaluate the performance of our approach on the corpus.

### Experiments settings

Following the previous work (18), a simple tokenizer (32) was used for tokenization and the Stanford CoreNLP Tool (33) was employed for sentence splitting, part-of-speech tagging and lemmatization. The BLLIP parser (34) with ‘GENIA + PubMed’ model was employed to obtain the syntactic parsing trees, and the dependency structures were extracted by the Stanford CoreNLP Tool. For inter-sentence level relation extraction, the Mallet MaxEnt classifier (35) was adopted because of its universality and effectiveness for classification problems.

The parameters for the CNN-based model at intra-sentence level were tuned on the development dataset. To train the CNN-based model, the AdaGrad algorithm (36) was applied to fine-tune *θ* with a learning rate of 0.002 and a mini-batch size of 64. As it is infeasible to perform full grid search for all hyper-parameters, we empirically set *w *=* *5, *v *=* *9, *n*_1_* *=* *300, *n*_2_* *=* *1500, *λ* = 10^−4^, *p* = 0.3. The look-up table of ***T*** was initialized by GloVe (37) and the dimension of *d*_0_ was set to 300 accordingly. Due to the huge size of the GloVe vocabulary (∼2 M words), we only kept the words that occurred in the CDR corpus. If the word existed in GloVe, we used its corresponding embedding for initialization; otherwise, we took a random embedding vector as the substitute. Eventually there were <20 K words in the vocabulary (the CNN code can be found at https://github.com/JHnlp/CNN_CDR). Other parameters in the model were initialized randomly.

All experiments were evaluated by the commonly used metrics Precision (*P*), Recall (*R*) and harmonic *F*-score (*F*).

### Experimental results

Table 4 shows the performance of the CID relation extraction at intra-sentence level on the test set with gold standard entity annotations using different features. From the table, we can observe that only using the contextual features, the final performance of *F*-score is able to reach as high as 54.8%. This suggests that contextual features are effective for intra-sentence level. Likewise, the model based on dependency features performs comparably with the one based on contextual features in terms of the *F*-score. This is probably because of its capability of representing the more general semantic relations between entity mentions. When combining contextual and dependency features, our system achieves the best *F*-score of 57.2% implying that dependency features and contextual features are complementary to each other.

**Table 4.** The performance of the CNN-based model on the test dataset at intra-sentence level

Methods	*P*	*R*	*F*
Contextual	54.8	54.9	54.8
Dependency	51.3	57.5	54.2
Contextual + dependency	59.7	55.0	57.2

Table 5 shows the overall performance of the CID relation extraction at both intra- and inter-sentence levels on the test set using gold entity annotations. During relation merging, the results from both intra- and inter-sentence level were fused into document level. The PP step was conducted following the relation merging step in order to improve the performance.

**Table 5.** The overall performance on the test dataset

Methods	*P*	*R*	*F*
Inter-sentence level	51.9	7.0	11.7
Intra-sentence level	59.7	55.0	57.2
Relation merging	60.9	59.5	60.2
Post-processing	55.7	68.1	61.3

From Table 5, we can find the performance of inter-sentence level is quite low. This is probably because the CID relations at inter-sentence level spans several sentences and thus have much more complex structures which the traditional features could not capture effectively. Merging the relations from mention level into document level can improve the *F*-score to reach as high as 60.2%. After the PP stage, the *F*-score can further arrive at 61.3%. However, the PP step would dramatically improve the recall at the expense of the significant decrease in the precision.

**Figure 4.**
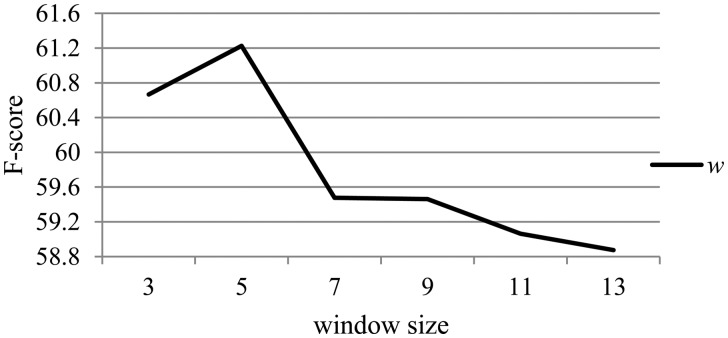
The effect of the hyper-parameter w on the development dataset.

Since *θ* was tuned on the development dataset, we need to evaluate the effects of the hyper-parameters with different values. The hyper-parameter *w* and *v* are mainly taken into consideration. Figure 4 depicts the effect of the hyper-parameter *w* related to the contextual information on the performance of the relation extraction at intra-sentence level on the development set. When *w* is changed, all other hyper-parameters remain the same as described in the section ‘Experimental settings’.

From Figure 4, we can observe that the performance rises first along with the increase of *w*, and reaches the best performance when *w* equals 5. Then the performance decreases as *w* is further increased. This is probably because the context around entity mentions within a window size of 5 is most suitable to describe their relations, while redundant information of the context would be detrimental to the identification of relations. Moreover, the more the context information is leveraged, the more the noise is introduced, as well as the larger the size of parameters in *θ* which should be learned, leading to the need of bigger corpus.

**Figure 5.**
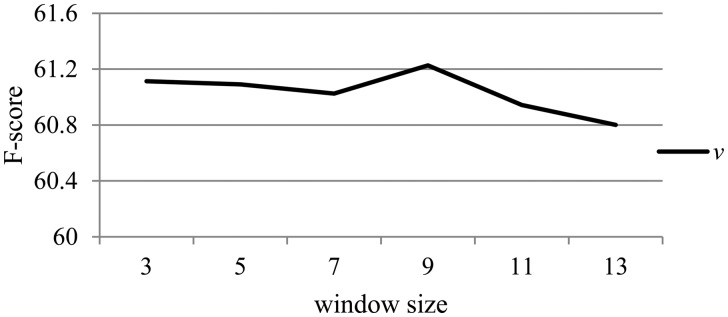
The effect of the hyper-parameter *v* on the development dataset.

Similarly, Figure 5 illustrates the effect of the hyper-parameter *v* related to the dependency information with different values on the development dataset. When *v* is changed, all other hyper-parameters remain the same as described in the section ‘Experimental settings’.

From Figure 5, we can observe that there is slight difference with the increase of *v*. This is probably because the dependency path can robustly reflect the direct syntactic relations between words, and the CNN-based method could grasp this kind of information effectively. However, the performance drops when *v* is larger than 9. It is probably because leveraging overmuch dependency information would be prone to induce some noise, and lead to the parameters being too large to learn with the small corpus.

## Discussion

In this section, we first present the error analysis by examining the errors of our results, then we perform the comparison between other relevant systems and ours.

### Error analysis

To understand why the task is challenging, we have closely examined the errors and grouped the error reasons as follows:

1. Lack of discourse inference: the relations at inter-sentence level were expressed spanning multiple sentences with a long distance, while the traditional linguistic features such as bag-of-words could not grasp the distant relation precisely. Therefore, discourse analysis including discourse inference and co-reference resolution should be needed for the inter-sentence level relation extraction.
2. Incorrect classification: the CNN-based method for intra-sentence level could represent the semantic features in a higher abstract way to represent more relative associations between entities, however, this would also introduce some noise to the system. For instance, in the sentence *Although the oral administration of class IC drugs, including****pilsicainide****, is effective to terminate****atrial fibrillation****, careful consideration must be taken before giving these drugs to octogenarians.* (PMID: *24653743*), our system wrongly extracted the relation between ***pilsicainide*** (C042288) and ***atrial fibrillation*** (D001281).
3. Inconsistent annotation: our experimental results show that some false positive relations we extracted were true and should actually be annotated in the corpus following the annotation guideline while they were actually not. For instance, our system found a CID relation between *METH* (D008694) and *hyperactivity* (D006948) in the document of PMID: 16192988. It is surprising that the relation was not annotated in this document, while it was actually annotated in other documents such as PMID: 15764424 and PMID: 10579464. This is probably because of the limited IAA of the CID relations as described in the section ‘Dataset’.
4. Rule-based extraction error: in the PP stage, heuristic rules were supposed to improve the recall by finding likely relations when none could be extracted by our system. Nevertheless, the rule-based approach was too aggressive to extract relations and would drop the precision significantly.

### Comparison with relate works

In the following, a comparison between our work and the relevant works is performed (18, 20–22, 30, 38, 39). Note that only the systems that reported their performance with gold standard entity annotations are selected in order to eliminate the influence of the accumulated errors introduced by different named entity recognition tools. Table 6 shows the performance of each system on the test dataset using gold standard entity annotations. We mainly divide the different methods into three groups as follows: the rule-based methods, the ML methods without additional resources, and the ML methods using external knowledge bases (KBs).

**Table 6.** Comparisons with the related works

Methods	System	Description	*P*	*R*	*F*
ML without KB	Ours	CNN	59.7	55.0	57.2
		CNN+ME	60.9	59.5	60.2
		CNN+ME+PP	55.7	68.1	61.3
	Zhou *et al.* (30)	CNN	41.1	55.3	47.2
		LSTM	54.9	51.4	53.1
		LSTM+SVM	64.9	49.3	56.0
		LSTM+SVM+PP	55.6	68.4	61.3
	Gu *et al.* (18)	ME	62.0	55.1	58.3
	Xu *et al.* (20)	SVM	59.6	44.0	50.7
ML with KB	Alam *et al.* (39)	SVM+KBs	43.7	80.4	56.6
	Xu *et al.* (20)	SVM+KBs	65.8	68.6	67.2
	Pons *et al.* (21)	SVM+KBs	73.1	67.6	70.2
	Peng *et al.* (22)	SVM+KBs	68.2	66.0	67.1
		Extra training data+SVM+KBs	71.1	72.6	71.8
Rule based	Lowe *et al.* (38)	Heuristic rules	59.3	62.3	60.8

From the table, we can find that the rule-based system, i.e. Lowe *et al.* (38), achieved a competitive performance with the *F*-score of 60.8% when compared with ML methods. However, the construction of their handcrafted rules is costly and time-consuming as well as domain dependent, which almost took over a period of 2 weeks (38).

ML methods with various kinds of features have shown a promising capability of extracting CID relations (18, 20, 30). Xu *et al.* (20) reported their performance with a feature-based SVM model, and only utilizing the linguistic features enabled them to reach an *F*-score of 50.7%. Our previous work (18) was based on a ME model with rich linguistic features, and the *F*-score could reach as high as 58.3%. Particularly, Zhou *et al.* (30) proposed a hybrid method which is the most relevant system to our approach. They incorporated a tree kernel-based SVM and an LSTM network to extract CID relations at sentence level. Their kernel-based model was aimed to capture the syntactic structures, while their LSTM model was supposed to generate the semantic representations. In (30), they reported multiple results of each sub-step of their method. From the table, we can find that their method reached 53.1% when only using the LSTM. When combining the LSTM with the SVM, their performance was improved to 56.0%. After employing heuristic rules in the PP stage their performance can be further improved. Though the PP step helped them to promote their performance to 61.3%, it significantly decreased their precision. In addition, they also tried a CNN model for comparison, but their CNN method only reached the performance of 47.2%. Compared with Zhou *et al.* (30), our CNN-based method exhibits a promising ability for the relation extraction at sentence level, with the *F*-score as high as 57.2%, rivaling the systems with rich linguistic features (18, 20, 30).

Apart from the above systems, methods using KB features have been proved to be more effective (20, 21, 22, 39). Alam *et al.* (39) leveraged knowledge features as well as various kinds of linguistic features. Xu *et al.* (20) leveraged an SVM model with various knowledge-based features. In particular, they took advantage of the relation labels of chemical and disease pairs in the CTD (4) KB, from which the CDR corpus was mainly generated. In (20), it reported that the features from KB could contribute nearly 11% of the *F*-score to their performance. Pons *et al.* (21) also used prior knowledge about chemicals and diseases to generate knowledge-based features with a fine tuned SVM classifier. They utilized the relation cues between chemicals and diseases in a large knowledge database which also includes the curated information in CTD. Peng *et al.* (22) proposed a rich-feature approach with SVM to extract CID relations. Their features included statistical features, linguistic knowledge features, and domain resource features. Furthermore, they augmented their approach with 18 410 external curated data in CTD as additional training data to further improve the performance. In (22), it reported that the KB features and the extra training data can contribute nearly 8.33 and 4.7% to their *F*-score, respectively.

Though features based on KBs especially on the CTD can yield a remarkably high performance because of the abundant manually refined information, our approach presented in this article still exhibits a promising improvement in precision as well as in recall. Compared with the knowledge-based systems, our approach would be more universal and easier to apply.

## Conclusion and future work

This paper describes a supervised learning approach to automatically extract CID relations by using a ME model and a convolutional neural network model for extracting inter- and intra-sentence level relations, respectively. Our study shows that the combination of the two models is effective on the CID relation extraction task. We believe our method is robust and can be readily adopted for other relation extraction tasks without much manual efforts for domain adaptation.

Our research on deep learning exhibits promising results for relation extraction in the biomedical literature. Nevertheless, more work needs to be done to further improve the system performance. In future work, we plan to include neural network models with richer representation such as recursive neural network and incorporate more knowledge from publicly available databases in a distant supervision fashion in order to achieve better results.

## Funding

This work was supported by the National Natural Science Foundation of China [grant numbers 61331011, 61373096 and 61673290].

*Conflict of interest*. None declared.
